# Editorial: Promoting Oral Health in Early Childhood: The Role of the Family, Community and Health System in Developing Strategies for Prevention and Management of ECC

**DOI:** 10.3389/fpubh.2021.716695

**Published:** 2021-07-19

**Authors:** Rahul S. Naidu, June H. Nunn, Bhavna Pahel, Richard Niederman

**Affiliations:** ^1^School of Dentistry, The University of the West Indies, St. Augustine, Trinidad and Tobago; ^2^School of Dental Science, Trinity College Dublin and Dublin Dental University Hospital, Dublin, Ireland; ^3^Private Practice in Paediatric Dentistry, Fort Mill, SC, United States; ^4^Epidemiology and Health Promotion, New York University, New York City, NY, United States

**Keywords:** early childhood caries, prevention, public health, strategies and solutions, primary dental care

Early childhood caries (ECC), has been defined as the presence of one or more decayed, missing due to caries, or filled tooth surfaces in any primary teeth in children under 6 years of age and is recognised as a global public health problem (1), affecting almost half of preschool children around the world (2). If untreated, ECC can lead to negative health impacts including acute infection, need for emergency care, and economic impacts for the family and society (3).

Paediatric primary care practitioners including family physicians and paediatricians generally have much earlier contact with families of infants and preschool age children than do dental practitioners (4). This is due to uptake of immunisation services, and infant health checks, in both developed and developing countries. Across the health system these primary care practitioners, therefore, play a critical role in early childhood oral health including caries risk assessment, providing preventive care (if needed) with silver diamine fluoride and fluoride varnish, and referral to dental care providers. A recent review of oral interventions for expectant mothers and those with young children, found maternal oral health education, caries risk assessment and appropriate referrals by non-dental professionals, can lead to a sustained reduction in early childhood caries (5).

## Education and Training

Policy statements from the American Academy of Paediatric Dentistry (AAPD) recommend: (1) referral by the child's primary care physician based on risk assessment, no later than 12 months of age, and (2) this assessment could be a routine component of new and periodic examinations by medical practitioners (6, 7). The World Health Organisation (WHO) also recommends that primary care teams and community health workers should understand the key risk factors for ECC, and how to identify them (3). However, in a recent review it was found that paediatricians frequently had limited knowledge of oral health and prevention in young children (8). The reasons given for this varied from barriers to education and training, time constraints, and lack of clear referral pathways.

Training should, therefore, involve early clinical signs of dental caries, recommended age of first dental visit, aetiology of dental caries and use of silver diamine fluoride and fluoride varnish. In a national survey of paediatric specialty trainees in the UK, it was found that although three quarters of the respondents agreed that paediatricians could assess oral health, nearly all felt that their training in oral health was insufficient (9). This indicates a need for oral health training in medical programmes, which enables competency in giving evidence-based preventive advice and support for families with preschool age children. Such training could be incorporated into the curriculum for general paediatric training. Inter-professional training at the undergraduate level may improve awareness of professional roles and facilitate interaction and communication between healthcare teams involved in child health. This requires development of competencies in knowledge and confidence in delivering oral health advice, for all paediatric primary care providers. Improvement in knowledge, confidence and practise towards children's oral health has been achieved through inter-professional education in graduate programmes (10). Sequentially, implementation includes awareness of the problem and proposed solutions, acceptance of information, application in practise and adapting to local health needs.

There is a perception still that attendance at a dental practise is more symptom-led than for medicine, which is more focused on preventive care for this age-group. It has been reported that most family physicians and paediatricians would refer a child with high caries risk for dental care but few would routinely refer a child at low risk for a first dental visit (11), suggesting some confusion regarding the importance of the dental home for young children. Dental teams also need to ensure clear and easy referral pathways from primary healthcare teams and non-dental health professionals. This has been achieved with children at high caries risk and in need of treatment (12).

## Research Topic Articles

In the context of the health system, two papers in this collection explored the effect of preventive oral care within state funded public health progammes. With a focus on fluoride varnish utilisation at medical well-child visits, Meyer and Danesh investigated inter-professional collaboration among dental and non-dental primary care providers and the impact of the COVID-19 pandemic on preventive care. They found quarterly fluoride utilisation rates and dental visits decreased significantly during the pandemic, highlighting the importance of inter-professional collaboration to provide access and preventive oral health services at physician offices.

Using machine learning pathways involving cluster analysis and cumulative dental cost curves, Peng et al. analysed utilisation patterns, service delivery models and cost effectiveness of preventive oral care among Medicaid insured children over 9 years. This revealed distinct clinical cost and utilisation patterns, and the need for preventive strategies that differentiate between specific subpopulations.

In the clinical setting, preventive oral care in early childhood is dependent on effective communication between the dental team and families with young children. In this context, Yuan et al. explored interactions between the dental professional-parent-child triad. Using conversation analysis, they identified three sequential phases of communications: social talking, containing worries, and task-focusing to develop strategic alliances for prevention and management of ECC.

In advocating for national upstream and downstream oral health promotion strategies Sitthisettapong et al. proposed a model for community-based prevention in Thailand. This model recommends an inter-professional, three-tiered approach to ECC prevention and management. The first tier is community education and use of fluoride toothpaste. The second tier is regular examination and early intervention. The third tier is the non-invasive use of atraumatic restorative treatment (ART, with or without silver diamine fluoride) all linked to effective monitoring systems.

The studies in this collection add to research that can inform the development of preventive strategies for ECC, as well as protocols/guidelines for all primary care teams that promote good outcomes for oral health in early childhood.

## Author Contributions

RNa and JN developed the manuscript, which was critically revised by RNi and BP. All authors contributed to the article and approved the submitted version.

## Conflict of Interest

The authors declare that the research was conducted in the absence of any commercial or financial relationships that could be construed as a potential conflict of interest.
